# Evaluation of the Antifungal Activity of *Mentha x piperita* (Lamiaceae) of Pancalieri (Turin, Italy) Essential Oil and Its Synergistic Interaction with Azoles

**DOI:** 10.3390/molecules24173148

**Published:** 2019-08-29

**Authors:** Vivian Tullio, Janira Roana, Daniela Scalas, Narcisa Mandras

**Affiliations:** 1 Department Public Health and Pediatrics, Microbiology Division, University of Turin, 10126 Turin, Italy; 2 Area Servizi Alla Ricerca Polo Agraria e Medicina Veterinaria, University of Turin, 10095 Turin, Italy

**Keywords:** essential oils, *Mentha x piperita*, “Mentha of Pancalieri”, azoles, antifungal activity, yeasts and dermatophytes, synergism

## Abstract

The promising antimicrobial activity of essential oils (EOs) has led researchers to use them in combination with antimicrobial drugs in order to reduce drug toxicity, side effects, and resistance to single agents. *Mentha* x *piperita*, known worldwide as “Mentha of Pancalieri”, is produced locally at Pancalieri (Turin, Italy). The EO from this *Mentha* species is considered as one of the best mint EOs in the world. In our research, we assessed the antifungal activity of “Mentha of Pancalieri” EO, either alone or in combination with azole drugs (fluconazole, itraconazole, ketoconazole) against a wide panel of yeast and dermatophyte clinical isolates. The EO was analyzed by GC-MS, and its antifungal properties were evaluated by minimum inhibitory concentration (MIC) and minimum fungicidal concentration (MFC) parameters, in accordance with the CLSI guidelines, with some modifications. The interaction of EO with azoles was evaluated through the chequerboard and isobologram methods. The results suggest that this EO exerts a fungicidal activity against yeasts and a fungistatic activity against dermatophytes. Interaction studies with azoles indicated mainly synergistic profiles between itraconazole and EO vs. *Candida* spp., *Cryptococcus neoformans,* and *Trichophyton mentagrophytes*. Thus, the “Mentha of Pancalieri” EO may act as a potential antifungal agent and could serve as a natural adjuvant for fungal infection treatment.

## 1. Introduction

With the wide use of synthetic and semi-synthetic antimicrobial drugs, their advantages and disadvantages have been highlighted over the years, including the spread of drug-resistant pathogens, and research has focused on the use of natural products as useful antimicrobial tools [1]. Currently, there is evidence that essential oils (EOs) may exert remarkable biological activities against viruses, bacteria, fungi, and parasites [1,2]. Furthermore, the promising antimicrobial activity of EOs has led researchers to use them in association with antimicrobial drugs in order to reduce toxicity, side effects, and microbial resistance. Several EOs are generally recognized as safe, do not accumulate in the liver or kidneys, can stimulate the immune system [3,4,5], and cause no resistance, since microbes are unable to adapt to their heterogeneous structures [6]. *Mentha x piperita* L. (peppermint) EO is one of the most widely produced and consumed EOs. Data show that peppermint EO and its main components (menthol and menthone) display antimicrobial effects, but their mechanism of action has still not been elucidated [7,8,9,10]. Near Turin (Piedmont, Italy), in Pancalieri, *Mentha x piperita* (Huds) var. Officinalis (Sole), form Rubescens (Camus) (Lamiaceae) is produced locally. This mint is known worldwide as “Mentha of Pancalieri” or “Mint Italo-Mitcham”. In herbal medicine, it is used in the form of the green plant that is dried for conservation or the EO obtained from steam current distillation of the whole plant (not shredded) [11]. The cultivation and distillation processes of this plant represents a secular tradition. Thanks to its high quality and uniqueness, the EO from “Mentha of Pancalieri” is actually considered by experts to be one of the best peppermint EOs in the world. Due to the continuous increase in fungal infections and the development of azole-resistant fungal strains, in our research, we assessed the antifungal activity of “Mentha of Pancalieri” EO against a wide panel of yeast and dermatophyte clinical isolates. Furthermore, the possible synergistic interaction of the EO with azoles, commonly used drugs in fungal infection treatment, was evaluated. Lastly, the anti-dermatophytic activity of the main components of the EO (menthol and menthone) and their interaction with azoles were assessed.

## 2. Results

The phytochemical composition of the “Mentha of Pancalieri” EO was confirmed to be rich in oxygenated monoterpenes (menthol 41.7%; menthone 21.8%), in accordance with data from the European Pharmacopoeia 8th Ed. (Table 1).

The “Mentha of Pancalieri” EO was found to exert good inhibitory activity against all tested fungal strains (Table 2), in comparison with azole drugs (Tables 4–6).

Notably, this EO exerted the most remarkable antifungal activity against *Cryptococcus neoformans*, displaying the lowest minimum inhibitory concentration (MIC) and minimum fungicidal concentration (MFC) values (Table 2, range 0.06–0.125%, *v*/*v*). Higher antimicrobial activity was also detected against dermatophytes (MIC = 0.125%, *v*/*v* for *Microsporum* species) and other non-*Candida* yeasts, such as *Saccharomyces cerevisiae*, and *Pichia carsonii* (MIC range 0.125–0.5 and 0.125–0.25%, *v*/*v*, respectively). It should be noted that the EO also showed non-negligible activity against *Candida krusei* and *C. glabrata*, yeasts that are often resistant to conventional drugs. Based on the MFC results, the “Mentha of Pancalieri” EO was found to display fungicidal activity against yeast cells (MIC = MFC) and fungistatic activity against dermatophytes. In fact, MFC values for dermatophytes were found to be one or two concentrations higher than MIC values.

The two main components of the “Mentha of Pancalieri” EO, menthol and menthone, were also evaluated against dermatophytes (Table 3). The results indicated that menthol possessed much lower MIC values (range 0.06–0.25%, *v*/*v*) against dermatophytes, in particular, against *Trichophyton mentagrophytes* (MIC = 0.06%, *v*/*v*) than menthone (MIC = 0.5–1%, *v*/*v*) and EO (MIC = 0.125–0.5%, *v*/*v*).

No significant differences in inhibitory concentrations were found between azole-susceptible (S) and azole-resistant (R) strains (Table 4 and Table 5), with similar MIC values observed in the presence of the “Mentha of Pancalieri” EO.

**Figure 1:**
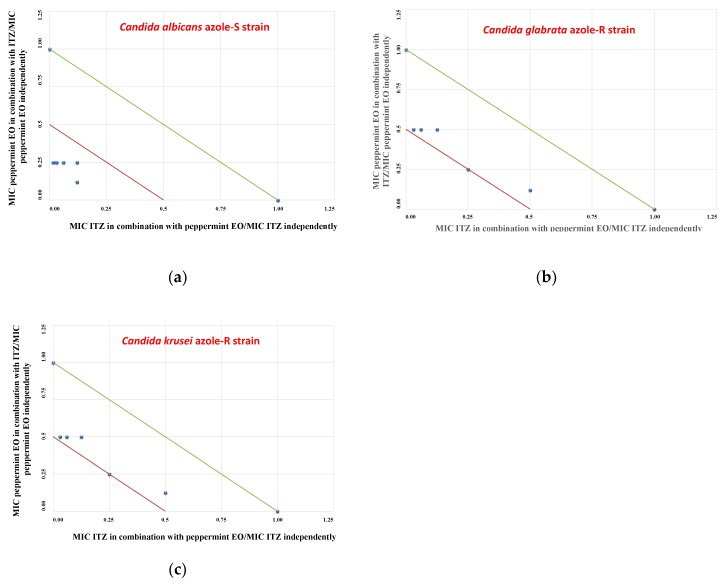
Isobolograms of synergistic interactions: plots of the “Mentha of Pancalieri” EO and ITZ against *Candida* spp. (azole-susceptible/resistant strains). ITZ FIC data are drafted on the x-axis, while EO FIC values are drafted on the y-axis: (**a**) isobologram plot of EO and ITZ against the *Candida albicans* azole-S strain; (**b**) isobologram plot of EO and ITZ against the *Candida glabrata* azole-R strain; (**c**) isobologram plot of EO and ITZ against the *Candida krusei* azole-R strain.

By chequerboard testing, binary combinations of fluconazole (FLC) with the “Mentha of Pancalieri” EO were found to be additive (0.5 < FICI ≤ 1; FICI = fractional inhibitory concentration index) against either both azole-S *C. albicans* and azole-S *C. glabrata* strains (Table 4). Notably, when synergistic or additive effects were not observed, no antagonism was reported, since binary mixtures of FLC/peppermint EO yielded indifferent effects on the azole-R *C. krusei* strain (FICI = 2). Notably, the combinatorial effects between itraconazole (ITZ) and the “Mentha of Pancalieri” EO were found to be synergistic (FICI ≤ 0.5) against all *Candida* spp. strains (Table 4). Moreover, the isobologram profiles confirmed synergistic associations between ITZ and the “Mentha of Pancalieri” EO (Figure 1a–c).

**Figure 2:**
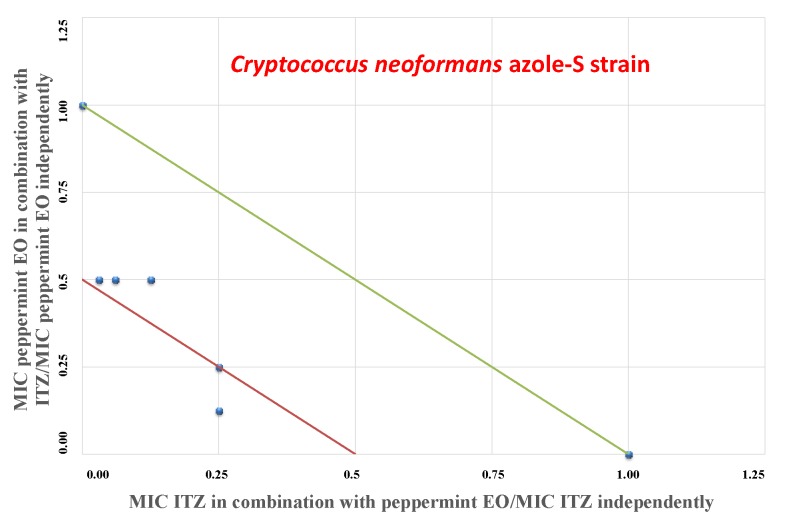
Isobologram of synergistic interactions: plot of “Mentha of Pancalieri” EO and ITZ against the *Cryptococcus neoformans* azole-S strain. ITZ FIC data are drafted on the x-axis, while EO FIC values are drafted on the y-axis.

In accordance with the FIC index values described for the *C. neoformans* azole-S isolate, the “Mentha of Pancalieri” EO was found to exert a synergistic profile (Table 5, FICI ≤ 0.5), with a decrease in the MIC, as expressed by the isobologram in Figure 2. On the contrary, concerning the *C. neoformans* azole-R strain, the binary combination of ITZ/peppermint EO yielded additive effects (FICI = 0.62).

**Figure 3:**
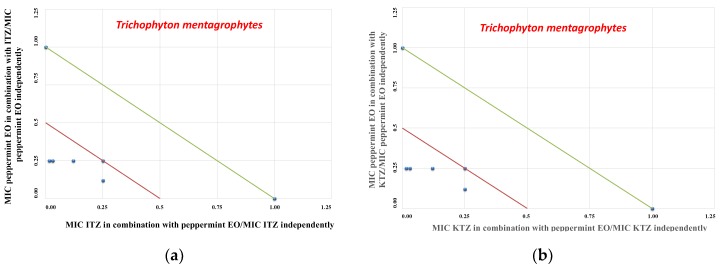
Isobolograms of synergistic interactions: plots of “Mentha of Pancalieri” EO and ITZ or KTZ against *Trichophyton mentagrophytes*. ITZ/KTZ FIC data are drafted on the x-axis, while EO FIC values are drafted on the y-axis: (**a**) isobologram plot of EO and ITZ against *T. mentagrophytes*; (**b**) isobologram plot of EO and KTZ against *T. mentagrophytes.*

By chequerboard testing, binary combinations of ITZ/KTZ with the “Mentha of Pancalieri” EO were found to be synergistic (Table 6, FICI ≤ 0.5) against *T. mentagrophytes*, as represented by the corresponding isobologram in Figure 3a,b. Conversely, indifferent interactions of ITZ/KTZ and “Mentha of Pancalieri” EO binary mixtures were recorded against either *Microsporum canis* or *M. gypseum* strains (Table 6, 1 < FICI < 4). Furthermore, results about menthol and menthone in combination with ITZ or KTZ indicated only an additive activity (FICI = 0.75–1) of these components against *T. mentagrophytes* (Table 7).

## 3. Discussion

To our knowledge, this is the first time that antifungal activity of the “Mentha of Pancalieri” EO, a local variety of *Mentha x piperita* (“Mint Italo-Mitcham”), alone and/or in combination with antifungal drugs has been studied. In fact, literature data mainly concern *Mentha x piperita* (peppermint). The leaves of peppermint (fresh and dried) and its EO are very well known herbal medicinal products that have been widely used for a long time. They represent a popular remedy inside and outside European countries due to their antispasmodic, choleretic, and carminative properties [14,15]. Some studies have demonstrated that *Mentha x piperita* EO possesses antimicrobial properties and antioxidant activity [15]. In addition, *Mentha x piperita* EO is capable of inhibiting the production of *Candida* and other microbial biofilms [16,17,18], reducing the amount of exoproteins associated with *Staphylococcus aureus* virulence at subMIC concentrations in a dose-dependent manner [19]. In this study, “Mentha of Pancalieri” EO exhibited higher antimicrobial activity towards *C. neoformans*, *S. cerevisiae*, and *P. carsonii* and displayed good antimicrobial activity towards *C. krusei* and *C. glabrata*—species that are often resistant to conventional drugs. This antifungal activity towards yeast strains is probably consistent with that of *Mentha x piperita* L. EO that is able to carry out anticandidal activity with consequent cell “death” by decreasing the amount of ergosterol and producing intracellular acidification following PM-ATPase inhibition [4]. Recent studies have shown that *Mentha x piperita* L. EO inactivates *S. cerevisiae*, a species that deteriorates fruit juices through the disorder of several physiological functions, i.e., enzymatic activity, efflux pump activity, and early apoptosis [20].

The activity of the “Mentha of Pancalieri” EO was also assessed against some etiological agents of dermatophytosis (*T. mentagrophytes*, *M. canis* and *M. gypesum*), usually treated with azoles. “Mentha of Pancalieri” EO showed higher activity against these dermatophytes than azole drugs and displayed fungistatic activity (Table 2 and Table 6), in agreement with a study by Ibrahim et al. with *Mentha x piperita* L. EO [8].

The antimicrobial activity of “Mentha of Pancalieri” EO can be mainly related to menthol and/or menthone, the main compounds contained in the EO [10]. However, this activity may be due to a possible synergy between all of the EO components [15,16]. In fact, while our MIC results for menthol (Table 3) indicated a better activity of this component against dermatophytes in comparison with EO in toto, the MIC values of menthone for *Microsporum* spp. (Table 3) were found to be twice as high as those observed with the EO in toto.

Since drug combinations represent a promising strategy for the development of new antifungal strategies, in order to overcome drug resistance, limit the side effects and toxicity of drugs, and increase therapeutic efficacy [16,21], in this research, we assessed the combined effect of “Mentha of Pancalieri” EO and three antifungal azoles: FLC, ITZ, and KTZ. Our data show a synergistic interaction between ITZ and “Mentha of Pancalieri” EO against *Candida* spp., *C. neoformans,* and *T. mentagrophytes*, with a noteworthy reduction in the concentration of azoles at the MIC. However, our FICI results for menthol and menthone in combination with ITZ or KTZ indicated only an additive activity (FICI = 0.75–1; Table 7) of these components against dermatophytes in comparison with EO in toto, suggesting once again that the activity of an EO is due to the phytocomplex that expresses its effectiveness better.

Taken together, these results suggest that “Mentha of Pancalieri” EO exhibits high antifungal activity and may have potential effectiveness as an adjuvant in association with ITZ or KTZ. Further studies on the mechanism of action of synergistic combinations as well as in vivo preclinical models are needed to predict the potential use of these data in clinical settings.

## 4. Materials and Methods

### 4.1. Essential Oil

Commercial “Mentha of Pancalieri” EO, obtained from the fresh leaves of *Menthax piperita* (Huds) var. Officinalis (Sole), form Rubescens (Camus) (Lamiaceae) by steam distillation, was purchased from Erbe Aromatiche Essenzialmenta, Pancalieri (Turin, Italy; http://www.essenzialmenta.it/). The composition of “Mentha of Pancalieri” EO was analyzed by GC-MS at the Drug Science and Technology Department (University of Turin, Italy) with a Supelcowax capillary column (Supelco, Bellefonte, PA, USA) as previously described [22]. Menthol (2-isopropyl-5-methylcyclohexanol (≥98% purity) and menthone [2-isopropyl-5-methylcyclohexanone, ≥97% purity] were purchased from Sigma-Aldrich (Milan, Italy) and were used without further purification. For susceptibility testing, EO and its components were dissolved in ethanol (1:2.5), and diluted (1:20) up to 2% (*v*/*v*) in RPMI-1640 medium with L-glutamine and without sodium bicarbonate (Sigma-Aldrich), as previously described [23]. Then, 0.165 M of morpholinepropanesulfonic acid (MOPS) (Sigma-Aldrich) and 0.2% glucose were added to the EO/components solutions. The final pH was 7.0. The EO and components were protected from light and humidity and maintained at 4 °C until just before use [23].

### 4.2. Antifungal Agents

Fluconazole (FLC; F8929), itraconazole (ITZ; I6657), and ketoconazole (KTZ; K1003) powders (≥98% purity by HPLC) were obtained from Sigma-Aldrich. FLC solution was prepared in sterile distilled water, whereas ITZ and KTZ solutions were prepared in 100% DMSO (Sigma-Aldrich). The solutions were stored at −20 °C until just before use [21].

### 4.3. Fungal Strains

Sixteen Candida spp. (C. albicans, C. glabrata, C. krusei, C. parapsilosis, C. tropicalis, C. valida, C. lusitaniae, C. norvegensis), 15 non-Candida spp. clinical strains (C. neoformans, S. cerevisiae, Kloekera japonica, P. carsonii, Sporobolomyces salmonicolor), and five dermatophyte clinical isolates (T. mentagrophytes, M. canis, M. gypseum) were tested (Table 2). C. albicans ATCC 90028 and C. glabrata ATCC 90030 were also included.

Yeast identification was conducted by the API ID32C system (BioMérieux, Rome, Italy). Then, strains were maintained at –80 °C in Microbanks™ (Pro-Lab Diagnostics, Neston, UK) and cultured twice on Sabouraud dextrose agar (SDA, Oxoid, Milan, Italy) at 35 °C for 72 h before the assays. Dermatophyte identification was carried out by macroscopic and microscopic observations of the colonies and reproductive structures after 15 days of incubation on Mycobiotic agar at 25 °C (Merck, KGAA, Darmstadt, Germany) [23,24]. Molds were stored in SDA at 4 °C until use. For susceptibility testing, non-germinated conidial suspensions were prepared as previously described [23].

### 4.4. In Vitro Antifungal Susceptibility Assays

Fungal strains were assayed for susceptibility to “Mentha of Pancalieri” EO and its components and to azoles (FLC, ITZ, KTZ) by a broth microdilution method, in accordance with the CLSI guidelines (CLSI M27-A3 and M27-S4 [25,26] for yeasts and CLSI M38-A2 [27] for dermatophytes), with some modifications for the EO and components [21,23]. Tween 80 (Sigma-Aldrich) (final concentration 0.001%, *v*/*v*) was employed to enhance EO solubility without precluding fungal proliferation. The final EO concentration ranged from 1% to 0.0019% (*v*/*v*), and the maximum ethanol concentration was 1.5% (*v*/*v*). Growth controls consisted of RPMI-1640 medium, and RPMI-1640 contained 1.5% (*v*/*v*) ethanol with no inhibitory effect on fungal growth at this concentration [23]. Blastoconidia yeast suspensions and dermatophyte conidial suspensions were prepared to yield the final inocula of ~1.5 × 10^3^ CFU/mL and ~1.5 × 10^4^ CFU/mL, respectively [21,23]. Microdilution plates were set up, as previously described [21,23], and yeast strains were incubated at 35 °C for 24 h, whereas dermatophytes were incubated at 30 °C for 7 days. All fungal species were tested in triplicate. The MIC values of FLC, ITZ, and KTZ were determined visually as the lowest concentration of drug that produced complete inhibition (dermatophytes) or a significant reduction (≥50% inhibition, yeasts) in growth in comparison with the control. MIC values of EO were considered to represent the lowest concentration with no visible growth. The MFC values were determined by inoculating 10 μL from non-turbid wells on SDA agar plates incubated for 3 (yeasts) and 4 (dermatophytes) days at 30 °C or at 35 °C (*Cryptococcus* sp). The MFC was defined as the lowest concentration resulting in no growth or fewer than three colonies to obtain approximately 99.9% killing activity on subculture from the MIC [21,23].

### 4.5. Checkerboard Assays and Evaluation of the Fractional Inhibitory Concentration Index

A two-dimensional checkerboard with serial two-fold dilutions below the MIC to 2× the MIC of each compound was performed in a 96-well microtiter plate, as previously described [21,28]. Microorganism suspensions were joined to each well containing mixtures of azole drug/EO or its components. FICI values were calculated according to the following formula [28]: FICI = FICa + FICb = MICa in combination/MICa tested alone + MICb in combination/MICb tested alone, where MICa and MICb are the MICs of azole and the EO or the component used alone. Synergy and antagonism were defined by FICI values of ≤0.5 and > 4, respectively. A FICI value between 0.5 and 1.0 was interpreted as additive, whereas a value between 1.0 and 4.0 was interpreted as indifferent [28].

### 4.6. Isobolograms

Data from the checkerboard assays are expressed graphically by isobolograms, as previously described [21,28]. The FIC values of the azole drug are reported on the y-axis, whereas FIC values of EO are reported on the x-axis. The straight line that joins the intercept points is the line of additivity (FICI = 1). Below this line, we can find the additive area (0.5 < FICI ≤ 1) and synergistic (FICI ≤ 0.5) effects, respectively. FICI values above the straight line correspond to indifferent (1 < FICI < 4) or antagonistic (FICI > 4) interactions [28].

### 4.7. Data Analysis

Results were obtained from three independent experiments performed in triplicate and expressed as modal results.

## 5. Conclusions

“Mentha of Pancalieri” showed good antifungal activity against several important fungal pathogens, including *C. krusei* and *C. glabrata*, which are often resistant to conventional antimicrobial agents.

Interaction studies with azoles indicated mainly synergistic profiles between ITZ and this EO vs. *Candida* spp., *C. neoformans,* and *T. mentagrophytes.* The EO of “Mentha of Pancalieri” contains mainly menthol (41.7%) and menthone (21.8%), which contribute to its good antifungal activity, according to guidelines of other countries and the European Pharmacopeia. However, our results are difficult to compare with literature data because of the different methodologies used in experimental procedures and the different *Mentha x piperita* L. EO compositions. For example, the EO acquired from the dried leaves of *Mentha x piperita* in Brazil contains 51% linalool as the main compound [29], probably due to the different cultivation conditions. It is very important that mint cultivation and EO production are strictly controlled, because it is recognized that the chemical composition of mint EOs, as well as all EOs, can be influenced by many factors including humidity, nutrients, and temperature. From this point of view, “Mentha of Pancalieri” has a guaranteed controlled composition and, consequently, high quality, which makes it an effective product for exploitation.

In conclusion, “Mentha of Pancalieri” EO may act as a potential antifungal agent and may serve as a natural adjuvant for fungal infection treatment. Hence, this EO may represent a more effective and less toxic alternative to antifungal drugs. Further research is necessary to confirm these data.

## Figures and Tables

**Table 1:** Phytochemical composition of “Mentha of Pancalieri” essential oil (EO) in comparison with the European Pharmacopeia indications for *Mentha x piperita* (Lamiaceae) essential oil.

Main Components	Mentha of Pancalieri EO (Peak Area %) ^1^	Eur. Ph. 8th ed. (Peak Area %)
Limonene	1.8	1–3.5
1,8-Cineole	5.3	3.5–8
Menthone	**21.8 ^2^**	**14–32**
Isomenthone	1.5	1.5–10
Menthil-acetate	4.8	2.8–10
Isopulegole	0.16	max 0.2
Menthol	**41.7 ^2^**	**30–55**

^1^ Relative percentages of individual components expressed as the % peak area relative to the total composition of the EO obtained by the GC-MS analysis ^2^ Main components.

**Table 2:** MIC ^1^ and MFC ^2^ ranges of “Mentha of Pancalieri” essential oil towards yeasts and dermatophytes from clinical and ATCC strains.

Species	Isolates (no.)	MIC Range (%, *v/v*)	MFC Range (%, *v/v*)
***Candida* spp.**			
*Candida albicans*	ATCC 90028	0.5	1
*Candida albicans*	6	0.5–1	0.5–1
*Candida glabrata*	ATCC 90030	0.5	0.5
*Candida glabrata*	2	0.5	0.5–1
*Candida krusei*	1	0.25	>1
*Candida parapsilosis*	1	0.5	0.5
*Candida tropicalis*	1	1	1
*Candida valida*	2	0.25–0.5	0.25–0.5
*Candida lusitaniae*	1	0.5	0.5
*Candida norvegensis*	2	0.25–0.5	0.25–0.5
**non*-Candida* spp.**			
*Cryptococcus neoformans*	7	0.06–0.125	0.06–0.125
*Saccharomyces cerevisiae*	4	0.125–0.5	0.25–1
*Pichia carsonii*	2	0.125–0.25	0.125–1
*Sporobolomyces salmonicolor*	1	0.5	1
*Kloekera japonica*	1	0.5	0.5
**Dermatophytes**			
*Trichophyton mentagrophytes*	2	0.5	>1
*Microsporum canis*	2	0.125	>1
*Microsporum gypseum*	1	0.125	>1

^1^ MIC = minimum inhibitory concentration; ^2^ MFC = minimum fungicidal concentration.

**Table 3:** MIC ^1^ and MFC ^2^ ranges of menthol and menthone, the main components of “Mentha of Pancalieri” essential oil towards dermatophytes clinical strains.

Dermatophytes (no.)		Mentha of Pancalieri EO (%, *v/v*)	Menthol (%, *v/v*)	Menthone (%, *v*/*v*)
*Trichophyton mentagrophytes* (2)	MIC	0.5	0.06	0.5
	MFC	>1	0.25	1
*Microsporum canis* (2)	MIC	0.125	0.25	0.5–1
	MFC	>1	1	>1
*Microsporum gypseum* (1)	MIC	0.125	0.25	1
	MFC	>1	1	1

^1^ MIC = minimum inhibitory concentration; ^2^ MFC = minimum fungicidal concentration.

**Table 4:** MIC ^1^ and FICI ^2^ of the “Mentha of Pancalieri” EO plus fluconazole or itraconazole against azole-S/R *Candida* spp. strains.

Antifungals		*C. albicans* Azole-S Strain ^3^	*C. glabrata* Azole-S */R ** Strain^3^	*C. krusei* Azole-R Strain ^3^
*Mentha of Pancalieri* EO	MIC (%, *v*/*v*) alone	0.5	0.5	0.25
Fluconazole	MIC (µg/mL) alone	0.12	4 *	64
	FIC of EO	0.5	0.5	1
	FIC of FLC	0.12	0.12	1
	**FICI**	**0.62**	**0.62**	**2**
	**Interpretation**	**ADD ^4^**	**ADD**	**IND**
*Mentha of Pancalieri* EO	MIC (%, *v*/*v*) alone	0.5	0.5	0.25
Itraconazole	MIC (µg/mL) alone	0.5	2 **	2
	FIC of EO	0.12	0.25	0.25
	FIC of ITZ	0.12	0.25	0.25
	**FICI**	**0.24**	**0.5**	**0.5**
	**Interpretation**	**SYN ^4^**	**SYN**	**SYN**

^1^ MIC = minimum inhibitory concentration; ^2^ FICI = fractional inhibitory concentration index; ^3^ breakpoints determining susceptibility/resistance to fluconazole (FLC) and itraconazole (ITZ) according to the CLSI document: FLC S ≤ 0.125 µg/mL and R ≥ 4 µg/mL (*C. albicans*), FLC R ≥ 64 µg/mL *C. glabrata)*; *C. krusei* is basically resistant to FLC; ITZ S ≤ 0.125 µg/mL and R ≥ 1 µg/mL (*C. krusei*, *C. glabrata*) [12,13] ^4^ ADD: additive; IND: indifferent; SYN: synergy. *= azole susceptible (S) strain; ** = azole resistant (R) strain.

**Table 5:** MIC ^1^ and FICI ^2^ of the “Mentha of Pancalieri” EO plus itraconazole against azole-S/R *Cryptococcus neoformans.*

Antifungals		*C. neoformans* Azole-S Strain	*C. neoformans* Azole-R Strain
*Mentha of Pancalieri* EO	MIC (%, *v*/*v*) alone	0.06	0.06
Itraconazole	MIC (µg/mL) alone	0.5	2
	FIC of EO	0.12	0.5
	FIC of ITZ	0.25	0.12
	**FICI**	**0.37**	**0.62**
	**Interpretation**	**SYN ^3^**	**ADD**

^1^ MIC = minimum inhibitory concentration; ^2^ FICI = fractional inhibitory concentration index ^3^ ADD: additive; SYN: synergy.

**Table 6:** MIC ^1^ and FICI ^2^ of the “Mentha of Pancalieri” EO plus itraconazole or ketoconazole against dermatophytes.

Antifungals		*Trichophyton mentagrophytes*	*Microsporum canis*	*Microsporum gypseum*
*Mentha of Pancalieri* EO	MIC (%, *v*/*v*) alone	0.5	0.125	0.125
Itraconazole	MIC (µg/mL) alone	0.5	1	1
	FIC of EO	0.25	1	1
	FIC of ITZ	0.12	0.03	0.125
	**FICI**	**0.37**	**1.03**	**1.125**
	**Interpretation**	**SYN ^3^**	**IND ^3^**	**IND**
*Mentha of Pancalieri* EO	MIC (%, *v*/*v*) alone	0.5	0.125	0.125
Ketoconazole	MIC (µg/mL) alone	2	4	2
	FIC of EO	0.25	1	1
	FIC of KTZ	0.12	0.015	0.015
	**FICI**	**0.37**	**1.015**	**1.015**
	**Interpretation**	**SYN**	**IND**	**IND**

^1^ MIC = minimum inhibitory concentration; ^2^ FICI = fractional inhibitory concentration index; ^3^ IND: indifferent; SYN: synergy.

**Table 7:** MIC ^1^ and FICI ^2^ of menthol and menthone plus itraconazole or ketoconazole against *Trichophyton mentagrophytes.*

Compounds	*Trichophyton mentagrophytes*	
Menthol	MIC (%, *v*/*v*) alone	0.06
Itraconazole	MIC (µg/mL) alone	0.5
	FIC of menthol	0.5
	FIC of ITZ	0.5
	**FICI**	**1**
	**Interpretation**	**ADD ^3^**
		0.125
Menthol	MIC (%, *v*/*v*) alone	0.06
Ketoconazole	MIC (µg/mL) alone	2
	FIC of menthol	0.5
	FIC of KTZ	0.5
	**FICI**	**1**
	**Interpretation**	**ADD**
Menthone	MIC (%, v/v) alone	0.5
Itraconazole	MIC (µg/mL) alone	0.5
	FIC of menthone	0.25
	FIC of ITZ	0.5
	**FICI**	**0.75**
	**Interpretation**	**ADD**
		0.125
Menthone	MIC (%, *v*/*v*) alone	0.5
Ketoconazole	MIC (µg/mL) alone	2
	FIC of menthone	0.5
	FIC of KTZ	0.5
	**FICI**	**1**
	**Interpretation**	**ADD**

^1^ MIC = minimum inhibitory concentration; ^2^ FICI = fractional inhibitory concentration index;^3^ ADD: additive.
